# Observations on the Cause and Prevention of Dental Caries

**Published:** 1908-12-15

**Authors:** J. Sim Wallace


### OBSERVATIONS ON THE CAUSE AND PREVENTION OF DENTAL CARIES.

BY J. SIM WALLACE, M.D., D.SC.GLASG., L.D.S., R.C.S.ENG.

Why are the teeth of the present generation so bad? This is a question which has puzzled the dental profession no less than the public generally, and with each successive generation the urgency of an answer to the problem has become more imperious. When the pathology of dental caries was fully elucidated it was thought by many that in this we had also the answer to the question. But it was not so. The question still remained unanswered, and the elaborate investigations which were undertaken since the pathology of the disease was established have practically all ended with negative results. I intend to show, however, that the solution of the problem is now, to all intents and purposes, an accomplished fact. For, indeed, the knowledge of the etiology of the disease which we now possess has enabled us to devise a means of preventing the disease.

The late Professor W. D. Miller defined dental caries as a "chemicoparasitical process, consisting of two distinctly marked stages, decalcification or softening of the tissue, and dissolution of the softened residue." If we make a little addition and say dental caries is a chemicoparasitical process dependent upon the undue lodgment of fermentable carbohydrates and acid-forming micro-organisms, we will find that this epitomises not only the pathology but also the etiology of the disease. It is now recognized that we are not to look merely to the existence of carbohydrates in the food as the cause of the disease but rather to those conditions which lead to their undue lodgment and consequent fermentation. I shall enumerate the conditions which favor the undue lodgment of carbohydrate food. These may be divided into two groups; (1) those resulting from the nature or constitution of the individual; and (2) those resulting from the nature or constitution of the food.

The first group includes peculiarities in the teeth themselves—*e.g.*, pits, abnormally deep fissures, abnormal shapes of teeth favoring the lodgment of food, and the size of the teeth. These may be regarded as in the main hereditary and they have been recognized as predisposing causes of caries ever since the pathology of the disease was established. But as these same peculiarities existed in previous generations and exist even at the present day in anthropoid apes they throw no light whatever on the question why the teeth of the present day are so bad.

The next constitutional conditions which favor the lodgment of food are certain developmental defects, pits, and grooves, such as result from hypoplasia of the enamel. This is rarely present in the temporary but occurs in the permanent set in about 6 percent. of children. As a rule hypoplasia is believed to result from some constitutional disease during the development of the enamel. I do not know of any statistics on the point, but perhaps hypoplasia may be rather more prevalent than it was in past generations on account of a greater amount of disease among children of the present day than in past generations or on account of more children affected with such diseases coming to maturity. If, however, this is claimed as a cause of the prevalence of dental caries it must be a very small factor indeed. Akin to this is the idea that on account of defective development of the teeth their molecular constitution may be in some way predispose them to decay, even though there is no visible defect in the enamel, and there has been a belief widely prevalent that the decreased amount of breast feeding may have seriously affected the constitution of the teeth in this way. Clinical evidence, however, does not appear to favor this idea very much, for the amount of caries in breast-fed children is almost as great as among the bottle fed children. Moreover, as the bottle feeding is more likely to be unduly prolonged, and so to affect the teeth after their eruption, even although there may be more caries among the bottle fed, it does not necessarily follow that it is due to defective constitution of the teeth.

These observations seem to be confirmed by a recent investigation by Mr. Ranulph B. Hunter, with regard to the teeth, of 500 school children. He gives the following statistics of the condition of the teeth. Breast-fed only: good, 44 percent.; moderate, 35 percent.; poor, 17 percent.. and bad, 5 percent. Artificial food only: good, 33 percent; moderate 43 percent.; poor, 16 percent.; and bad, 8 percent. From the above it will be seen that unless we omit the "moderate" teeth the comparison does not lend itself to any deduction of a practical nature.

Considering that bottle-feeding more frequently gives rise, or at least predisposes, to various serious troubles eventuating sometimes no doubt in malformed and crowded teeth, that is to say, giving rise to conditions favoring the undue lodgment of food, we are justified in saying that if the constitution of the teeth is affected at all it is by no means an important factor in the causation of caries at the present day. In a similar way we might refer to alterations or absence of the normal buccal secretions as predisposing to the lodgment of food, but as far as we know abnormalities of this kind are rare except during severe illness, *e. g.*, fever, and they may be dismissed as being of little or no importance in giving a clue to our original question.

The next constitutional factor to which we may refer as predisposing to undue lodgment of food is irregularity of the teeth. This factor has long been recognized as a fruitful predisposing cause of caries. Inasmuch as irregularities are not frequent in the temporary teeth this in no way accounts for the great prevalence of the disease in young children, nevertheless it is an important predisposing cause in the permanent teeth. As, however, the etiology of irregularities is a complex subject it may be sufficient to note that the abnormal arrangement of the teeth results from mouth-breathing, from general and prolonged ill health, from deficient general nutrition, and from insufficient development of the muscles of mastication, including the tongue.

So far we have only briefly considered some of those factors depending upon the constitution of the individual, and only the last throws any light on our question, foi irregularities of the teeth were not prevalent with our ancestors at least with our remote ancestors, so that this may in some measure be considered responsible for a certain amount of the increased prevalence of dental caries in adults, but inas much as irregularities of the teeth result largely from insufficient mastication and deranged nutrition our explanation would be very insufficient if we did not note the changes which have given rise to decreased mastication and deranged nutrition. We must, however, leave this in the meantime and refer to the more important branch of our subject —namely, to certain changes in the foodstuffs which tend to increase the lodgeability of the carbohydrates and acid-forming micro-organisms. The carbohydrate foodstuffs have undergone great changes. Cellulose in the natural or uncooked state is, as a rule, of a consistency which stimulates vigorous mastication, but in civilized countries the cellulose is cooked, softened, and often altogether extracted from the food, so that the detergent effect which this has in its natural state is almost completely lost. Indeed, it may be presumed that in the state it is frequently presented it helps to clog the crevices rather than to brush away the bacteria and fermentable food particles which may lodge about the teeth. In fact, the absence of cellulose in a form which should stimulate the pleasurable activity of efficient mastication practically nullifies the mechanical, the hydrodynamical and, to a certain extent, the chemico-physiological self-cleansing processes of the mouth.

The next carbohydrate to which we may briefly refer is starch. This when cooked or boiled becomes of a pasty nature, and it is easily converted into sugar which becomes rapidly fermentable. Starch, therefore, when taken in the food unaccompanied by food of a detergent nature is eminently fitted to lodge and undergo acid fermentation in the mouth.

Lastly, we may refer to sugar. Sugar has long been supposed to have a deleterious effect upon the teeth. The investigations, of Miller, however, tended to show in his opinion that it was to be considered as less harmful to the teeth than starch; and the fact, or at least the supposed fact, that children who consumed large quantities of sugar from the sugar-cane were relatively free from dental caries gave rise in the minds of most dentists to the idea the "sugar bogey" had been slain. And although most would admit that neither starch nor sugar would be beneficial, there was, of course, little use of talking ahout preventing children eating both sugary and starchy foods. Miller based his criticism largely on the fact that sugar was readily soluble and was therefore, "soon carried away or so diluted with saliva as to be rendered harmless." Clinical evidence, however, supports the idea that the sugars are more harmful than the starches, no doubt from the fact that in addition to inversion cane-sugar undergoes a mannitic fermentation forming a gummy substance which not only clings about the teeth, but also tends to catch other particles of food, and to retain them in contact with the teeth also. Secondly, sugar hampers the action of the saliva, and lastly the method of eating sugar in the form of bonbons causes a continuous supply of this fermentable material to be available for the acid-forming bacteria lodging in the crevices of the teeth. Moreover, concentrated sugar such as one gets in sweets has an irritating effect on the mucous membrane, and it is probable that the mucus secreted under these conditions may rather favor the retention of food particles than otherwise. With regard to the relative excellence of the teeth among the natives in the sugar-cane plantations, this is, of course, largely due to the fact that the fibrous cellulose of the cane stimulates the self-cleansing factors most thoroughly. I do not know whether cane or grape sugars are the most harmful; it seems milk to solid food is usually carried out by a few extracts from a recent authoritative text-book published "to aid the young practitioner" of medicine. So far as this is concerned the author, it will be granted, is strictly orthodox. After nine months, he says, "dilution of the milk is not necessary. Some solid food may be added in the shape of boiled bread or porridge, or pudding. These additions must be small in quantity at first." Then when the child is 12 months old he says, "The porridge may be made thicker and of coarser catmeal, A little potato and gravy or half an egg may be given once a day. Soft bread-and-butter or dripping may be taken." Now what happens when an infant hitherto accustomed to milk is given bread well soaked in milk? The first noticeable effect is that the infant gulps down the milk-soaked bread and milk without any attempt at retaining it in the mouth or mixing it with saliva. The starchy matter in the bread is therefore washed into the stomach without any insalivation, without any conversion of the starch, and without any preparation for digestion in the stomach. The physiological effect which the retention and mastication or gnawing of food in the mouth produce is practically lost, and the flow of digested juices in the stomach is correspondingly lessened. The palate is cheated, for large amounts of carbohydrates are washed rapidly past it, and instead of appreciating the amount of converted starch, or rather of unconverted starch, which the child has consumed, it craves for more of that very substance of which it has already consumed too much—in other words, it develops an abnormal craving for sugar. Further, by becoming habituated to swallowing solid food it soon loses that automatic mechanism which arrests solid food in the mouth till it has become liquified and prepared for deglutition. At a later stage, say about the thirteenth month, when the first temporary molars have taken their positions, what happens when the child is restricted to this soft diet? The previous troubles continue and the teeth get dirty and tender from want of use; later they become carious and the tenderness increases, while for the same reasons mastication is not performed, and so these troubles and others resulting therefrom become more or less thoroughly established. The child is pronounced constitutionally or hereditarily delicate, and a whole list of troubles are attributed to teething which in reality are the outcome of digestive derangement resulting from an erroneous system of dietary.

Now why should we not take a hint from Nature? Mother's milk when the child reaches nine months or a year does not become more solid, rather the reverse. Suppose we give cow's milk; why should we make it less diluted than it was before, seeing that the time is beginning to approach when the child will and can eat solid food and drink liquid water? The child has been accustomed for all the months it has existed to have its mother's nipple in its mouth (or an artificial substitute), and from this it has been able to express or suck liquid food. When it is determined to give the child solid food, why not let it get a solid piece into its mouth? In other words, why not let it have half a slice of bread, or better, perhaps, toasted bread and butter? No doubt the child feels that toast and butter is not its mother's breast, and it certainly subjects the toast to the influence of its teeth. It gnaws it and sucks it; the gnawing induces a flow of saliva and the ptyalin converts the starch. The child continues to suck much as it sucked its mother's breast, and its palate appreciates that it is actually sucking liquid out of the solid toast. Gradually the toast disappears, practically in the form of liquid, down the child's throat, thoroughly prepared for further digestion in the stomach. Before the fourteenth month, or at least before the first temporary molars erupt, true mastication, of course, is not performed. It is gnawing which is indulged in, and it certainly is indulged in by all children. Should they not be given the chance to gnaw solid food they will pick up the pieces of stuff they find on the floor and gnaw and suck them instead, unless they are supplied with a "comforter," which is perhaps nearly as bad and generally quite as dirty. After pretty certain, however, as a clinical fact that sugar as usually consumed, sweets, marmalade, jam, etc., is markedly harmful to the teeth, and as it appears to be necessary for medical men to tell their patients to "knock off" sugar for so many complaints we may soon have to petition the Chancellor of the Exchequer to put a heavy tax on sugar whatever our political opinions may be. Moreover, inasmuch as bread, which is the chief source of proteid among the poor, contains an excess of carbohydrates, the spending of money by this class on sugar is practically sheer waste from the point of view of physiology. This point should always be considered by those who belaud sugar on account of its cheapness.

Before the true part played by the foodstuffs was known it was, of course, impossible to prevent the disease; indeed, even to the present day all that is generally done is to advocate the tooth-brush and antiseptics after every meal, and this has not been altogether satisfactory by any means. It had come to be accepted that any ordinary meal of necessity left the mouth dirty and consequently that it had to be cleaned artificially; it was never supposed that meals which were physiologically correct actually cleaned the mouth. It was recognized that to prevent people eating carbohydrates or limiting them to those which did not ferment rapidly was out of the question and the idea that the food should be sufficiently hard to afford the teeth the exercise necessary for their vigorous development was not acted upon, probably because we were all aware that their development was completed before they cut the gums and in the case of the temporary teeth before the child had stopped sucking liquid milk. Now that we know the role of the foodstuffs in the etiology of caries is possible for us to show how the disease may be prevented, and although it has been said that people would not adopt the method we advocate, it is a fact that already many people have adopted it and where it has been commenced at a sufficiently early age the results have so far been absolutely perfect.

In dealing with the prevention of dental caries in children we must take things as we find them and this necessitates us considering the subject both when the teeth are still free from caries and when the teeth have already been attacked by the disease. We have seen that the teeth always erupt free from caries and in case of the first teeth we find also that they almost invariably cut the gums free from any developmental defects which might predispose to the undue lodgment of carbohydrate foods. I shall refer to the deciduous teeth more especially because certain predisposing causes such as irregularities and recession of the gums are absent and the natural crevices are relatively small. Moreover, we have no grounds for assuming that the natural instincts or habits of the young child have been in any way perverted as they frequently are in older children. We have seen from the etiology of caries that the all-important subject is the environment of the teeth and not their inherent structure. It behooves us, therefore, to consider the dietic habits of civilized children from the time that the teeth came through the gum. And first of all let us direct our attention to that period of transition from the toothless infant to the child with a complete set of deciduous teeth and correspondingly from the milk diet to the solid and varied diet for which the child's teeth and alimentary canal become so admirably adapted.

Now for the first six or nine months of a child's life it has been accustomed to extracting liquid from the mother's breast, and through all the ages of man's evolution nothing was mixed with this milk. When, with increasing age and concomitant changes, the first article of food was presented to the child it was given at a different time from the sucking of the milk. I think it must be obvious that in a state of nature it was utterly impossible to soak food in milk in order to effect the transition from mother's milk to the ordinary foods of children and adults. Moreover, there is no precedent which could in any way be interpreted as justifying the method of transition from milk to solid food which has been so universally adopted. I shall exemplify how the transition from the child has had its bread and toast to supplement its milk diet, say twice a day for a month or two, then other things may be added, such as rusks and milk puddings made sufficiently solid, and as there is not an excess of albumin in the milk (it having been diluted) boiled fish and chicken may be given in small amounts. I have had some little experience of this method of feeding infants and I can say most unhesiingly that coughing, choking or spluttering has been conspicuous only by its total absence. But this is not all; the desire for hard food remains. The teeth do not become tender nor the mouth dirty nor the teeth carious. The palate is not cheated and the desire for excess of food or sweets does not exist. The alimentary canal performs its functions in a natural and healthy manner, and by the age of two and a half years, when it has its full set of temporary teeth, the child can and may be allowed to eat practically any food which adults habitually consume.

Now before alluding to actual dietaries suitable for children after they have their twenty temporary teeth, it will be well to refer briefly to certain points in oral hygiene, for however simple it may be to arrange dietaries in such a way as to secure physiological cleanliness of the mouth, it is highly desirable to recognize the main features of the normal and natural processes by which physiological cleanliness is maintained, however complex these processes may be. For convenience in description we may consider the processes under different headings although they are more or less intimately associated with and dependent upon each other. I shall briefly allude to each.

Firstly, we have a *mechanical* process. This depends to a great extent on the physical consistency of the food. When the food is of a firm and somewhat fibrillar consistency it stimulates the pleasurable activity of efficient mastication.

Secondly, we have a *chemico-physiological* process. Food, when taken into the mouth stimulates a flow of saliva, and carbohydrate food, especially if slightly acid, and firm in consistency, stimulates the secretion of saliva rich in ptyaline. The mastication helps to incorporate the ptyaline in the food and the insoluble starch becomes converted more or less into soluble sugar which is ultimately swallowed in a liquid or nearly liquid form. This is, of course, the natural method of treating carbohydrate food, and in passing it may be said the physiological method of leaving the mouth free from carbohydrates at the end of a meal. It should be remembered, however, that much sugar hampers the action of the ptyaline.

Lastly, we have a *saprophytic* or *bacterial* process. In the whole history of man or animal the mouth has never been free from bacteria. And the bacterial flora of the mouth seems to pay an important part in its hygiene. There is no pepsin or other ferment in the mouth which can digest or liquify the various albuminous shreds which are apt to lodge between the teeth. But there are many of the mouth bacteria which have this power. In fact, these bacteria digest and liquify the albuminous shreds which lodge about the teeth and so allow of their dissolution. They give rise to a continual disintegration and removal of food particles and tend to keep the teeth clean at those very situations which are not kept clean by natural friction of the food, tongue and lips. I do not say that all the bacteria which may be in the mouth are beneficial, for even some of those which have the power of liquifying albuminous matter have also the power of producing acid when lodging food particles are of a starchy or sugary nature. The strictly liquifying mouth bacteria, however, seem to be quite innocuous to the teeth before caries is started, and if the dietary is arranged physiologically, these beneficial mouth bacteria are favored, while, on the other hand, the harmful—the acid-forming bacteria—are, in my opinion at least, prevented from proliferating. For it appears that when acid-forming bacteria produce a certain amount of acid, this acid itself arrests their further growth. Similarly weak acids in foods may be presumed to have a like effect on the acid-forming bacteria.

These being the most important factors in oral hygiene we may now refer to the dietaries of children. It must be evident that on the whole the dietary should be of a consistency demanding or stimulating efficient mastication. In other words porridge and milk puddings, soft white bread, and things of this sort, should not form a great part of the meal. As bread forms a considerable part of children's meals, the easiest way to insure a reasonable amount of hardness is to substitute crisp toast, or baked bread, or crusty bread rolls, for the plain white bread. It is, however, of little consequence how the meal is made sufficiently hard to stimulate mastication so long as it is done. I need not say more on the necessity of insisting on this point. For whether oral hygiene, digestion in the mouth, or digestion in the stomach is considered, the necessity for food which demands efficient mastication is obvious.

The next point to which I would refer is the necessity to have the meal *arranged*. It is not a matter of indifference whether we eat in the orthodox arrangement or in the reverse order; there are good physiological reasons for the arrangement of the meal as custom so generally prescribes. I do not intend, however, to go into this in detail. I only wish to mention that in a well-organized repast dessert always follows sweets. This is done, not because physiologists have told us it is right, but I think it is because sweets leave a sticky or clammy feeling about the mouth, while fresh fruit leaves a clean and refreshing flavor. Over and above this, fresh fruit not only makes the mouth feel clean, it actually does clean the mouth, as will be seen by referring to the essential requirements of oral hygiene.

We must bear in mind these two points: the necessity for food which demands efficient mastication and, secondly, the necessity for finishing the meal in such a way that the mouth will be left physiologically clean. Then consider actual dietaries, such as are usually found at the present day, and we find that they frequently violate one or more of the physiological requirements we have referred to. For example, the following is quite a common breakfast: Porridge and milk, an egg, bread and marmalade, and milk, tea or coffee. On the whole, this meal is distinctly too soft and on that account would rather discourage efficient mastication and stimulate simply swallowing the food. On this account the detergent effect which should accompany mastication is lost and the food is not properly insalivated or prepared for passage into the stomach. Moreover, it may be presumed also that the stomach is not properly prepared, for the reception of the food, for the mastication of food has an effect on the secretion of the gastric juices. Secondly, the meal being finished with bread and marmalade the action of the saliva is hampered by the presence of concentrated sugar, and the sticky or lodgeable nature of the food tends to establish all the requisite conditions for the destruction of the teeth. It may be asked what should be recommended rather than the breakfast which we have criticised. Well, a typical, somewhat similar and yet satisfactory breakfast would be: bacon or bacon and egg, baked or toasted bread, fresh fruit, *e. g.*, a slice of a melon or an apple, followed by tea or coffee. This might by some be regarded as too much. It is quite easy to reduce it by omitting the bacon or egg or both. And if, for any reason, the fresh fruit is not desired, it may possibly be omitted also without harm resulting, provided the tea or coffee is taken *after* the meal.

With regard to the next meal, the luncheon or dinner, I need not say much. The errors are similar to those already referred to in the breakfast, but generally not so pronounced, as it usually includes a piece of meat of some kind, and this, as a rule, stimulates at least a little mastication. Moreover, I have heard that it is becoming fashionable to provide toast or baked bread at this meal, and although this probably originates from the fact that people may always be presumed to be suffering more or less from indigestion, still from whatever motives we may welcome the change. The midday meal however, generally terminates with sweet puddings of some kind or another, and although they may not be so bad as bread and marmalade or jam for leaving the mouth unclean, they certainly are not cleansing, and ought therefore to be followed by fresh fruit. With regard to what children should be given to drink with, or rather after, this meal, I am strongly of opinion that it should be water and not milk, but the reasons for this are numerous and it is unnecessary to refer to them just now.

With regard to the last meal of the day. It generally resembles either the breakfast or the lunch and we need not say much about it. Sometimes it is, however, what we might call a purely vegetarian meal, consisting chiefly of milk or tea, bread and butter, jam, scones and cakes. Now a meal such as this is particularly objectionable, as you will observe from what we have said, but being the last meal of the day the harmfulness is augmented by the fact that the mouth has not the chance of being thoroughly cleansed from the remains of such a meal till the next morning. It has been observed by many dentists that the teeth of vegetarians appear to be subject to rapid decay and this has been my experience. It is not always so, and it is by no means necessarily so, for a vegetarian meal can be arranged physiologically just as a mixed meal can. If the meal is composed of baked bread, or toast and cheese, or ship's biscuits and butter, followed by an apple there will be no reason to expect the slightest harm to result to the teeth.

So far we have assumed that the teeth are free from caries but it very frequently happens that the disease has already commenced before prevention is thought of. When caries is not far advanced in the temporary teeth all that is required to be done is to have the carious teeth filled and at the same time initiate the necessary changes in the diet which will in future prevent further decay in the teeth. To fill teeth and not to correct the error which brought on the carries amounts to little more than to invite the patient to return for similar operations in six months or a year. It should be distinctly realized that dental caries is a very distinct sign of persistent dietetic error and it is the duty of the medical practitioner to correct this error in any child in which he finds dental caries. It should also be borne in mind that the filling of teeth does not increase the auto-purification of the buccal cavity, at least outside the radius of the filling itself. That, indeed, the mouth may be kept continually in a foul and dangerous state although the teeth are filled regularly every three months.

So far we have assumed that it was possible to restore the teeth to full functional activity, but very frequently it is not. The caries may have advanced so far as to have involved the pulp. In such cases it is practically impossible to restore the temporary teeth to a state of health. If an attempt is made to fill them they are very liable to remain more or less tender, if indeed something more serious does not happen. It is therefore necessary to extract such teeth, and when this is done the opposing tooth, whether sound or unsound, must also be extracted, for an unopposed tooth, at least in the molar region, is not self-cleansing. I may here say that quite frequently it is necessary to extract all the eight temporary molars. Under the direction of Mr. J. F. Coyler this method of treating children's teeth has been carried out for the last few years at the Royal Dental Hospital. Those who have seen the results have admitted that the children certainly do not suffer from what may at first appear a somewhat heroic method of treatment. I had the opportunity of seeing several cases where the treatment had been on these lines and the mouth had been converted, in my opinion, from a hot-bed of pathogenic bacteria to a self-cleansing cavity. I learn also that the children increase in weight and their general health improves. We can well understand this, for it is a common observation among dentists that the health of adults rapidly improves when several septic roots are removed from the mouth. Objections, of course, may be, and have been, raised to this method. No doubt it is not ideal, but the advantages are so marked that there can be no reasonable and sufficient objection under the conditions mentioned. This is true more especially from a general medical standpoint of view, for a mouth which is not physiologically clean is a constant and serious menace to health. It may be said that the extraction of teeth as indicated above can hardly be called preventing dental caries. But indeed it is, for inasmuch as the mouth is made self-cleansing, it prevents decay in the remaining teeth, it allows the child to use what teeth it has without discomfort or pain, it permits of a change of diet from sloppy to relatively hard food, and, moreover, it allows the permanent teeth to erupt in a clean and healthy mouth. These are all points of no small importance in the prevention of dental caries.—The *Dental Record*, October 1908.

